# Current and Future Uses of Continuous EEG in the NICU

**DOI:** 10.3389/fped.2021.768670

**Published:** 2021-11-03

**Authors:** Amanda G. Sandoval Karamian, Courtney J. Wusthoff

**Affiliations:** ^1^Division of Neurology, Children's Hospital of Philadelphia, Philadelphia, PA, United States; ^2^Division of Child Neurology, Lucile Packard Children's Hospital at Stanford, Palo Alto, CA, United States

**Keywords:** continuous electroencephalography, neonatal seizures, neonatal epilepsy, critically ill neonates, neurodevelopmental outcomes

## Abstract

Continuous EEG (cEEG) is a fundamental neurodiagnostic tool in the care of critically ill neonates and is increasingly recommended. cEEG enhances prognostication *via* assessment of the background brain activity, plays a role in predicting which neonates are at risk for seizures when combined with clinical factors, and allows for accurate diagnosis and management of neonatal seizures. Continuous EEG is the gold standard method for diagnosis of neonatal seizures and should be used for detection of seizures in high-risk clinical conditions, differential diagnosis of paroxysmal events, and assessment of response to treatment. High costs associated with cEEG are a limiting factor in its widespread implementation. Centralized remote cEEG interpretation, automated seizure detection, and pre-natal EEG are potential future applications of this neurodiagnostic tool.

## Introduction

Continuous electroencephalography (cEEG) is full-array EEG recording, typically using a minimum of eight electrodes, performed over an extended period to non-invasively assess brain function. It is one of the most widely used forms of neuromonitoring in newborns. By providing real-time information about brain function, including information about spatial localization of brain activity, cEEG can offer rich detail. Furthermore, cEEG is the gold standard method for the diagnosis of seizures in the neonatal period and is widely used for this. The current use of cEEG in the NICU can be understood within the context of its application for background assessment, seizure detection, and how it might be employed in combination with other modalities. There is increasing evidence for the benefits of cEEG in the NICU, with future directions to hone utility.

## Guidelines for Use of CEEG in Neonates

While cEEG previously had been limited to use in only highly specialized centers, it is increasingly advised as standard care for all NICUs. World Health Organization (WHO) Guidelines advise that all suspected neonatal seizures should be confirmed by EEG where available (1). This is because neonatal seizures are notoriously difficult to diagnosis reliably through clinical observation alone; EEG is required to confirm diagnosis before initiating pharmacologic treatment. Full-array EEG is the most accurate method for confirming that a clinically suspicious event is epileptic in origin, and the risk of performing EEG on a neonate is minimal (1). In most settings, that EEG will be cEEG, as prolonged recording greatly increases the diagnostic yield for paroxysmal events. In addition to the WHO endorsement of EEG for seizure diagnosis, video cEEG has been particularly recognized as useful. In the updated Neonatal Seizure Classification by the International League Against Epilepsy (ILAE), video cEEG is an integral part of the framework for the diagnosis of neonatal seizures; EEG recording is the first step in diagnosis of seizures in a critically ill neonate at risk of or with clinical suspicion for seizures (2).

Similarly, the American Clinical Neurophysiology Society (ACNS) guidelines recommend the use of cEEG to determine whether paroxysmal events in neonates are seizures (3). In addition, the ACNS guidelines specify that cEEG should be used to detect seizures in newborns who are at high risk for having seizures, including those with known or at high risk for acute brain injury (See Table 1) (3). In cases where neonates with treatment refractory seizures are intentionally placed in burst suppression with medications, EEG should be used to monitor the suppression of the EEG and to detect seizure recurrence (3). Beyond seizure detection, EEG is further recommended to assess for background abnormalities in neonatal encephalopathy as a prognostic tool (3). Recommended duration of monitoring is outlined by EEG indication in the ACNS guidelines: EEG background assessment requires a minimum of 1 h of recording to allow analysis of sleep-wake cycling, neonates at high risk for seizures should have 24 h of cEEG monitoring, and neonates with confirmed seizures should have cEEG until they have been seizure-free for at least 24 h (3).

**Table 1:** Indications for cEEG monitoring in neonates [adapted from Shellhaas, et al. (3)].

**Indication**	**Examples**
Differential diagnosis of paroxysmal events/suspected clinical seizures	Abnormal movements, automatisms, autonomic changes
High risk clinical conditions	Hypoxic ischemic encephalopathy Cardiac arrest Persistent pulmonary hypertension Extracorporeal membrane oxygenation Cardiopulmonary bypass CNS infection CNS trauma Intracranial hemorrhage, including high grade intraventricular hemorrhage Inborn errors of metabolism Arterial ischemic stroke Cerebral venous sinus thrombosis Cerebral malformations
Monitoring response to treatment	Pharmacological burst suppression
Prognostication	Judgement of severity in neonatal encephalopathy

Across each of these guidelines, there is consistent endorsement of cEEG use in neonates. At a minimum this should be to confirm diagnosis when seizures are suspected clinically; however, there are several recommended uses beyond seizure confirmation.

## Continuous EEG for Background Assessment

**Figure 1:**
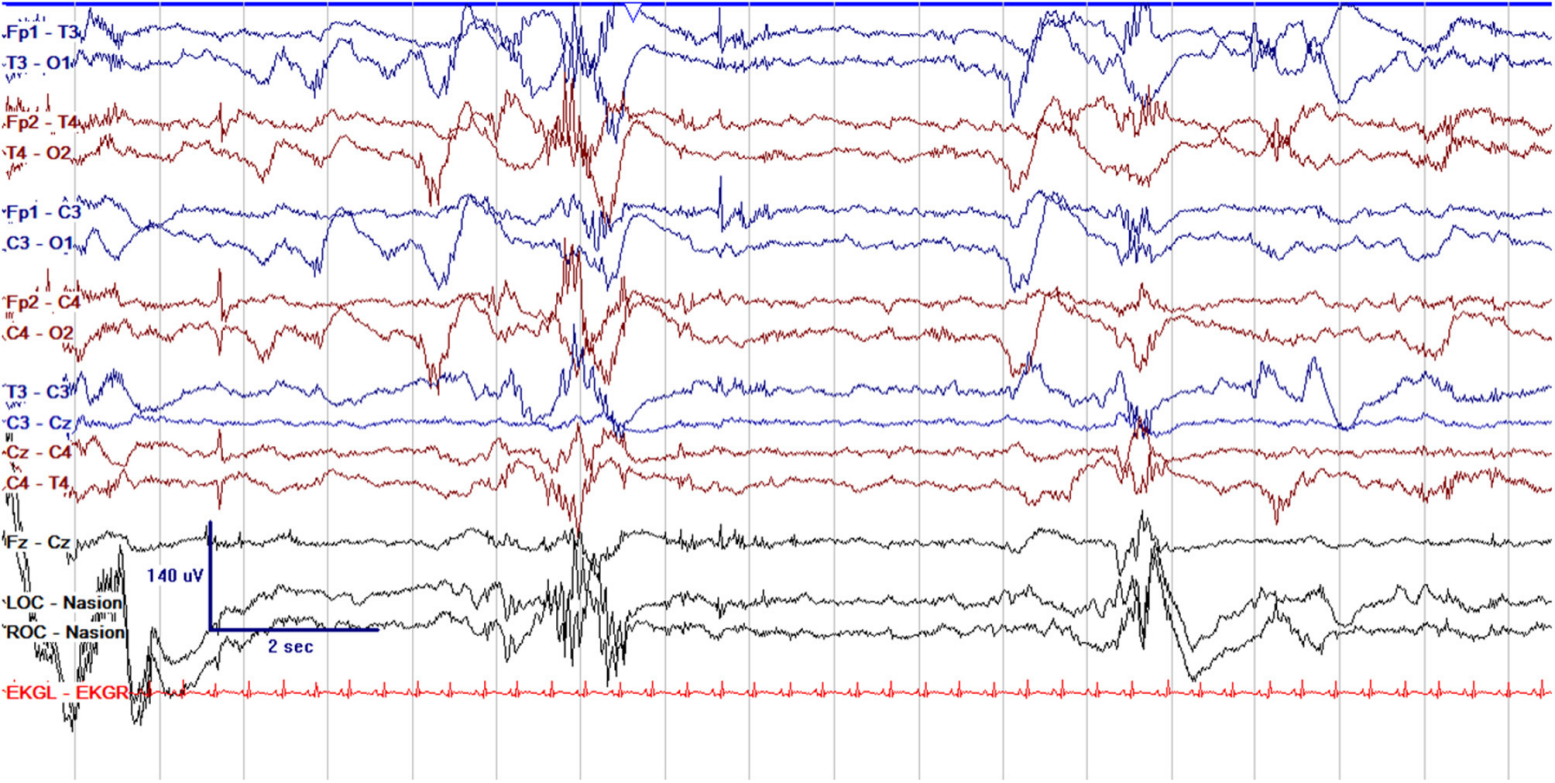
Normal cEEG in a preterm neonate, a 2 day old former 29 + 3 week gestational age (GA) [29 + 5 week post-menstrual age (PMA)] neonate with normal discontinuity for gestational age. In neonates with GA < 30 weeks, interburst intervals can be up to 35 s and <25 μV. In GA 30–33 weeks, interburst intervals can be a maximum of 20 s with voltage <25 μV. In 34–36 week GA neonates, interburst intervals can be up to 10 s and ~25 μV.

**Figure 2:**
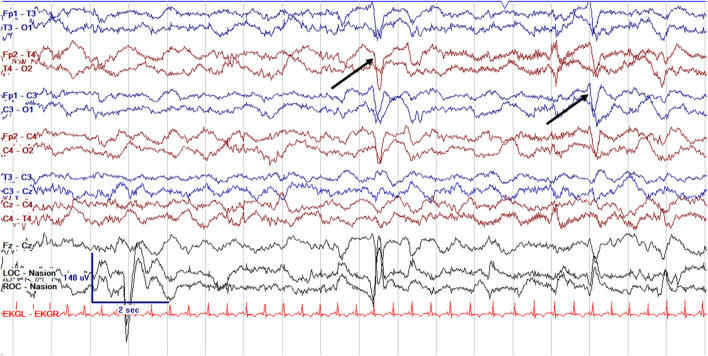
Normal cEEG in a term neonate in the awake state, a 3 day old former 39 + 0 week GA (39 + 3 week PMA) with normal continuous activity of variable voltage and frequency with normal graphoelements (encoche frontales–normal frontal sharps, arrows).

**Figure 3:**
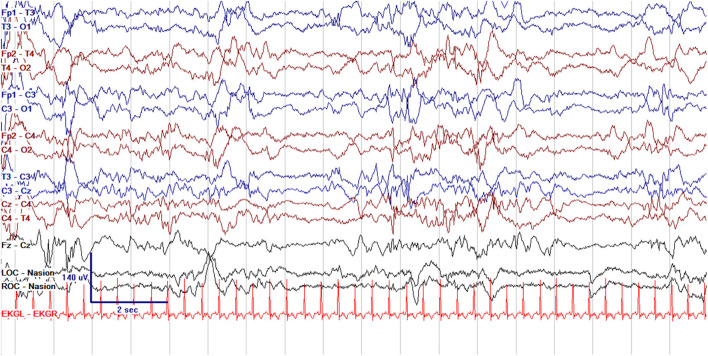
Normal cEEG in the same term neonate in quiet sleep, with normal discontinuity (trace alternans) with interburst intervals lasting up to 6 s and of voltage >25 μV.

**Figure 4:**
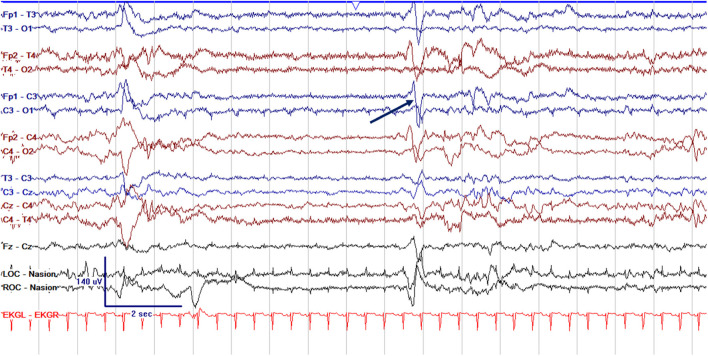
Abnormal excessively discontinuous cEEG background in a term neonate, a 1 day old former 39 + 0 week GA (39 + 1 PMA) with interburst intervals lasting >6 s with voltages <25 μV. Normal graphoelements, encoche frontales–normal frontal sharps, are seen (arrow).

**Figure 5:**
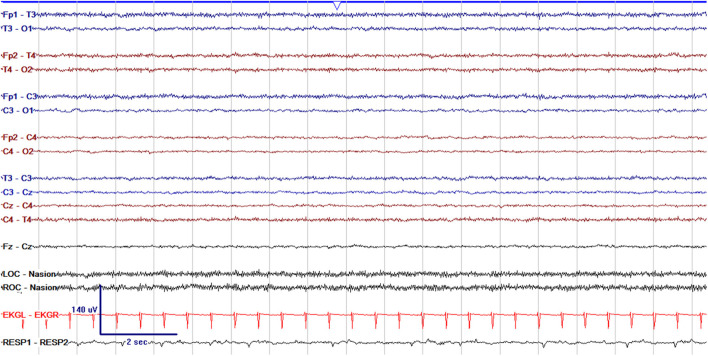
Severely abnormal cEEG background which is entirely suppressed with voltage <10 μV and featureless in a 1 day old former 37 + 6 week GA (37 + 7 week PMA) neonate.

**Figure 6:**
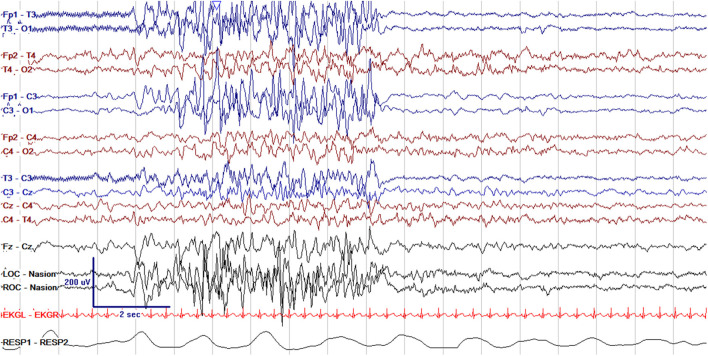
Abnormal asymmetric cEEG with discontinuous high amplitude bursts of epileptiform activity in the left hemisphere and more normal continuity and voltage in the right hemisphere in a 10 day old former 39 + 1 week GA (40 + 4 week PMA) with left hemimegalencephaly.

EEG monitoring allows for assessment of background brain activity in neonates as a reflection of brain health or injury. There are expected normal EEG backgrounds for awake and asleep states in neonates; this pattern matures with increasing gestational age (see Figures 1–3) with the criteria for normal discontinuity varying with gestational age (see Figures 1, 3) until reaching the expected continuous mixed activity seen at term equivalent age during wakefulness (see Figure 2) (4). There are named features called “graphoelements” which similarly indicate normal brain maturation and function when present and can indicate abnormality when absent or when not consistent with the neonate's conceptional age (4). Encoche frontales, which are normal frontal sharp transients, are an example of a normal neonatal graphoelement (see Figures 2, 4). When the EEG background is not as expected for age, it can indicate dysmaturity, acute injury, or other underlying abnormality. Commonly observed abnormal patterns include excess discontinuity, which describes a pattern with low-amplitude activity (<25 microvolts) sustained for longer than expected for age (see Figure 4). Burst suppression is a particularly worrisome pattern; this describes an invariant, extremely low voltage recording interrupted only by high amplitude bursts of activity, lacking any normal features. An entirely suppressed (<10 μV) and featureless tracing is another severely abnormal and concerning background pattern (see Figure 5). The term electrocerebral inactivity, formerly electrocerebral silence, is a term that can be applied only when specific technical requirements are met, which are different from the requirements for a standard neonatal cEEG recording (4). EEG background is typically symmetric; focal or hemispheric abnormalities should raise suspicion for a focal brain lesion (see Figure 6). In all cases, EEG background abnormalities can be useful for reflecting brain function at a single point in time. cEEG monitoring confers the additional advantage of revealing the trajectory of brain function over time, which may be more important. For example, in neonatal encephalopathy, it is common for the EEG background to be excessively discontinuous in the first hours after birth. However, early recovery, with improvement of discontinuity and gradual return to a normal background is associated with good prognosis. In contrast, an EEG which shows worsening discontinuity reflects an overall worsening of condition. In this way, cEEG for background assessment provides information about brain function at the current time, but also about how brain function is changing.

Evolution of cEEG background has been used to guide prognostication in Hypoxic Ischemic Encephalopathy (HIE). An early normal EEG background at 6, 12, and 24 h of life is predictive of a favorable outcome at 2 years of age (5). Conversely, abnormal background with sustained suppression or burst suppression at 72 h is predictive of death or severe disability in >90% (5–8). Prior to the widespread implementation of therapeutic hypothermia for neuroprotection in HIE, severe EEG background abnormalities at 6 h of age were associated with poor neurodevelopmental outcomes, while normal background at 6 h was associated with normal outcomes (5). Further, recovery of the background within 24 h was associated with improved outcomes (5). In the era of cooling, the time points at which EEG background is useful for prognostication have changed. Discontinuity on EEG for >30 s per min (equating to 50% or greater discontinuity) at 24 and 48 h of age was associated with MRI injury and worse neurodevelopmental outcome in term neonates with HIE who underwent therapeutic hypothermia (8). In another study, severe background abnormalities on EEG (discontinuous activity with interburst intervals 10–60 s, severe attenuation of background activity, no sleep-wake cycles, isoelectric EEG with activity <5 μV, or severe discontinuity with interburst interval >60 s) at 36 and 48 h of age was associated with severe injury on MRI and abnormal neurodevelopmental outcome in term neonates with HIE who underwent cooling (7). A large meta-analysis found that EEG background patterns of burst suppression, low voltage, and flat trace were the most predictive of abnormal neurodevelopmental outcome, with pooled estimates of sensitivity and specificity of 87 and 82% for burst suppression, 92 and 99% for low voltage, and 78 and 99% for flat tracing, however there was some variation in the definitions used for these background patterns across studies (6). The Total Body Hypothermia for Neonatal Encephalopathy Trial (TOBY) demonstrated a positive predictive value of severely abnormal aEEG for death or disability at 18 months of 56% (9).

EEG background can also be used to predict the occurrence of seizures, with higher sensitivity and specificity in models combining both clinical and EEG data (10–12). In a historical sample of 2,000 neonates, poor mental status with lethargy or coma and moderate to severe EEG background abnormalities were associated with seizures on EEG (10). A more contemporary sample of 210 neonates found that seizure prediction models combining clinical and EEG characteristics yielded a higher area under the curve of 83% compared to 66% with clinical variables alone and 76% with EEG variables alone (12). Neonates with clinically suspected seizures had higher risk compared to neonates with encephalopathy alone, and abnormal EEG background conferred higher risk for seizures, with more severe abnormalities having higher risk (excessively discontinuous background OR 7.10, burst suppression OR 19.45, and depressed/undifferentiated OR 27.78 compared to normal EEG) (12). By contrast, a normal EEG background and presence of sleep-wake cycling were associated with a low likelihood of seizures (OR 0.12 and OR 0.29, respectively) (12). In this sample of 210 neonates, only 2 with normal EEG background had seizures, and both had known brain injury (12). A different sample of 90 term neonates with HIE similarly found that initial EEG background was strongly associated with risk for seizures, with more severe background abnormalities conferring higher risk, independent of treatment with antiseizure medications prior to EEG (11). In this sample, however, 12% of neonates with normal initial EEG background did have seizures, though further clinical details of these subjects were not provided (11).

As the brain develops with increasing gestational age, the cEEG background demonstrates expected maturational changes, as seen in Figures 1–3. With brain maturation, there is expected evolution with increasing continuity, the appearance of sleep cycling, increasing hemispheric synchrony, and PMA-specific graphoelements (13). Brain dysfunction in preterm neonates may lead to altered rates of brain maturation with dysmaturity on EEG, a gap between the actual PMA of the neonate and the PMA suggested by the appearance of their EEG (4, 14, 15). cEEG allows the most accurate assessment of brain maturation, with visualization of graphoelements and synchrony (13, 16). EEG dysmaturity in pre-term infants has been associated with worse developmental outcomes at 12 and 24 months of age (15).

cEEG background can also be suggestive of specific diagnoses. Focal attenuation of one region or hemisphere is suggestive of focal dysfunction in that region, such as ischemia from an arterial stroke or venous sinus thrombosis. Focal epileptiform activity may be seen in cases of focal brain malformation, including extensive polymicrogyria or hemimegalencephaly (see Figure 6). Severe background abnormalities with burst suppression on EEG and refractory seizures in a neonate with encephalopathy of unknown etiology suggest a diagnosis of epileptic encephalopathy with likely genetic cause; mutations in the *KCNQ2* gene are the most common cause of neonatal onset genetic epilepsy and epileptic encephalopathy (17–19).

## Continuous EEG for Seizure Monitoring

**Figure 7:**
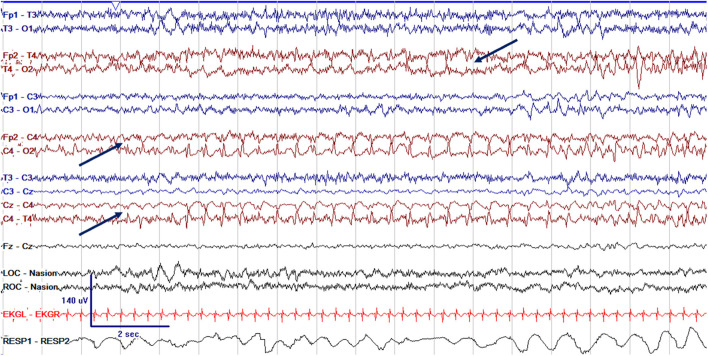
cEEG of a focal electrographic only seizure (arrows) arising from the right central region (C4), with spread to the right temporal region (T4) in a 3 week old former 38 + 4 week GA (41 + 4 week PMA) neonate.

Seizures are the most common neurological condition affecting neonates; cEEG is essential to their accurate diagnosis. Neonatal seizures affect 1–5/1,000 live births and have a wide range of etiologies (1, 20). Neonatal seizures are most often subclinical, or electrographic only, meaning they have no outward clinical signs and can be diagnosed solely by EEG (see Figure 7). An estimated 80–90% of seizures in neonates are subclinical, and those that initially have a clinical correlate frequently become subclinical after treatment (20–22). Electroclinical uncoupling, the phenomenon in which clinical seizures transition to subclinical seizures following administration of antiseizure medications, occurs in up to 58% after treatment with phenobarbital or phenytoin (20–22). For all these reasons, clinical diagnosis of neonatal seizures is challenging and often inaccurate; in a study in a large NICU network in Ireland, only 9% of true electrographic seizures were clinically diagnosed, while 73% of non-ictal events were incorrectly identified as seizures (23). A study with video review of events concerning for possible seizure identified only 50% correctly and demonstrated poor interrater agreement with a low Kappa of 0.2 (24).

Due to the high rates of subclinical seizures and inability to reliably diagnose seizures clinically in neonates, cEEG is required for accurate diagnosis. WHO, ILAE, and ACNS guidelines all advise that cEEG be used for diagnosis in neonates with clinical suspicion for seizures (1–3). Further, the ILAE and ACNS recommend cEEG for critically ill neonates at risk for seizures (see Table 1) (2, 3). Neonates at high risk for seizures should have 24 h of cEEG monitoring, and neonates with confirmed seizures should continue on cEEG for at least 24 h of seizure freedom (3). A recent large study of term neonates with acute symptomatic seizures further demonstrated the utility of cEEG for neonates at high risk for seizures. The rate of successful response to initial seizure treatment with an ASM was higher in neonates undergoing cEEG for seizure screening due to high-risk clinical conditions compared to neonates undergoing cEEG for confirmation of clinically concerning events for seizure (39% successful initial response to treatment vs. 18% response) (25). The suggested framework for implementation of therapeutic hypothermia for the treatment of HIE from the American Academy of Pediatrics also advises the use of EEG for seizure detection and monitoring and suggests that centers treating HIE have this resource available (26).

Ultimately, cEEG is most often used in the NICU to screen for and diagnose seizures. While further research is needed, there is evidence to suggest that neonatal seizures are independently associated with worsened developmental outcomes. In this way, cEEG to guide effective neonatal seizure diagnosis and treatment ultimately hopes to reduce the risk of adverse outcomes among neonates at risk. Neonatal seizures are most often acute symptomatic seizures caused by an acute brain injury, such as HIE or arterial ischemic stroke. In addition to the neurodevelopmental impact of the brain injury or condition causing the seizures, the seizures independently are associated with additional risk for further brain injury and worse neurodevelopmental outcomes. Neonates with seizures have more severe MRI brain injury and worse neurodevelopmental outcomes compared to those with a similar brain injury and no seizures, suggesting that the seizures themselves mediate further injury (27–30). Neonatal seizures lead to altered cerebral hemodynamics, increased cerebral metabolic demands above energy supply with decreased phosphocreatine to inorganic phosphate ratios, increased cerebral lactate and choline, and alteration of hippocampal neurons (31–34). Similarly, long-term outcomes are associated with presence of seizures and seizure burden: both clinical and EEG seizures are associated with poorer outcomes including lower IQ, cerebral palsy, and increased mortality (28–30). Higher electrographic seizure burden is associated with worse MRI injury and worse neurodevelopmental outcomes including motor and language delays, post-neonatal epilepsy, and cerebral palsy (27, 35–37). Given these neurodevelopmental consequences of EEG seizures in neonates, accurate diagnosis is essential. EEG is employed to guide treatment and in hopes of improving outcomes.

## Continuous EEG in Combination With Amplitude-Integrated EEG

While amplitude-integrated EEG (aEEG) is useful for bedside screening for seizures, cEEG is preferred for confirming a diagnosis of seizures. The use of both tools together allows teams to benefit from the particular strengths of each. Bedside monitoring with aEEG for seizures allows caregivers to directly review the simplified aEEG tracing without reliance on a neurophysiologist for interpretation. This can be an incredibly helpful tool. At the same time, cEEG remains necessary because aEEG used alone will miss some electrographic seizures and may lead to incorrect diagnosis of seizures in other cases. Neonatal seizures are often focal, brief, and of a slow frequency. Because the aEEG tracing filters out slow frequencies and compresses the time scale, very slow seizures with discharges at a frequency of <2 Hz or brief seizures lasting <30 s are not detected; seizures lasting 90 s appear for only 1.4 mm on the display (see Table 2) (38). aEEG also uses a limited number of channels which allows rapid application and bedside interpretation, but misses seizures that occur in locations not covered by the limited aEEG montage, including the frontal and posterior regions (39). The sensitivity and specificity of aEEG is also variable and highly dependent upon the skill level in interpretation, with reported sensitivity ranging from 40 to 85% and specificity ranging from 50 to 90% (40–47). Interrater agreement of aEEG seizure interpretation is also poor, with a Kappa value of only 0.3 (48). When considering the role of aEEG for neonatal seizures, it is best used as a screening tool when there is a high incidence of seizures and a high skill level of interpretation, with confirmation by cEEG (see Table 2) (40). In settings where cEEG is not readily available, aEEG and cEEG are best used in combination, with aEEG for seizure screening when there is a sufficiently high seizure prevalence and high skill level of interpretation and cEEG for seizure confirmation.

**Table 2:** Amplitude integrated EEG vs. continuous EEG use.

**aEEG uses**	**cEEG uses**
Background assessment	Background assessment
Seizure screening in populations with high pretest probability for seizures	Diagnosis and monitoring seizures
High skill level in interpretation	Slow frequency seizures, short duration seizures (will be missed by aEEG)
	Frontal and occipital seizures, or injury in those areas predisposing to seizures (will be missed by aEEG)

## Impact of CEEG in the NICU

Even as there is an association between seizure burden and adverse outcomes, there have remained questions about the benefits of cEEG use in the NICU, particularly when weighed against cost and other barriers. The balance of the potential benefits and costs may vary according to the resources at hand for a given center (see Table 3).

**Table 3:** Benefits and costs of cEEG.

**cEEG benefits**	**cEEG costs**
Tool for prognosis	EEG machines and equipment
Accurate diagnosis and monitoring of seizures	EEG electrodes
Decreased seizure burden for improved neurodevelopmental outcomes	EEG technicians
Decreased progression of seizures to status epilepticus	Neurophysiologist/epileptologist interpretation
Lower phenobarbital dosing and levels	Skin breakdown from electrodes
Decreased rate of discharges on an ASM	
Decreased length of stay	

Building evidence demonstrates that cEEG can be cost-effective and beneficial to short-term outcomes, such as reduced anti-seizure medication use and reduced length of NICU stay. In a cohort of neonates with HIE randomized to treatment of EEG seizures or clinical seizures, the use of cEEG to guide seizure management was associated with significantly lower seizure burden (median seizure burden of 449 vs. 2,226 s) and decreased time to treatment completion (mean time 79 vs. 170 min) (37). At another center, implementation of a cEEG-based neonatal status epilepticus protocol was followed by decreased progression of seizures to status epilepticus by 10%, and length of stay decreased by almost 10 days (49). There were also lower phenobarbital levels, indicating better targeted therapy with lower exposure to antiseizure medications (ASMs), which carry their own risks and adverse effects (49). An additional single-center study found that continuous EEG use increased electrographic seizure detection and at the same time decreased phenobarbital exposure, as well as decreased how many neonates were discharged on an ASM. This further demonstrates that even with the increased identification of seizures, cEEG allows for tailored therapy that minimizes overtreatment via accurate diagnosis (50). In a longitudinal study performed at a single center that assessed practices across three periods, when comparing cEEG to aEEG or routine EEG, there was improved seizure identification with cEEG as compared to aEEG or routine EEG. Furthermore, there was decreased risk of receiving an ASM with cEEG as compared to aEEG or routine EEG use (51). As cEEG monitoring is more widely used, mounting evidence shows that it is associated with improved short-term outcomes in the NICU.

## Future Uses of CEEG

Future work is needed to reduce barriers to cEEG implementation and improve the cost effectiveness of this technology (see Table 4). There are notable barriers to widespread implementation and use of cEEG (see Table 3). Hardware costs of EEG machines and electrodes represent a significant investment for settings with limited resources. There are also high personnel costs; skilled EEG technologists are needed to apply electrodes and trained neurophysiologists are required to interpret the EEG data. To further extend this resource, a larger institution can remotely interpret EEG for multiple hospitals using a digital central server with remote access. Implementation of remote cEEG at two hospitals affiliated with the Children's Hospital of Philadelphia identified electrographic seizures in 24% and impacted clinical care in 75% (52). This was feasible, effective and clinically significant care. This creates a centralized hub for EEG interpretation and management and increases access to the valuable resource of cEEG. Centralized, remote EEG interpretation is one strategy that neonatal neurology can employ to address health disparities by providing more equitable access to cEEG.

**Table 4:** Future directions for continuous EEG.

Centralized remote EEG interpretation
Automated seizure detection
Neonatal EEG caps
Fetal EEG

In addition to centralized EEG interpretation, automated seizure detection is another method that might allow further expansion of cEEG use in the future. Automated seizure detection algorithms applied to neonatal cEEG have wide ranges of reported sensitivities between 43 and 81% and specificities between 56 and 90% (53–59). The nature of neonatal seizures as being focal and often brief make automated seizure detection in neonates particularly challenging. With one method of automated neonatal seizure detection, the odds of detecting seizures increased with increasing seizure amplitude, duration, rhythmicity, and number of EEG channels involved (55). These algorithms are limited in their application to neonatal EEG due to artifacts and false-positive events which are frequently related to nursing care, including patting, along with respiratory artifacts, sweat artifacts, and increased rhythmicity in sleep (53, 55, 58, 59). Computer vision algorithms can be applied to video recording using dense optical flow estimation to reduce false positives in automated seizure detection (53).

Single-use or reusable neonatal EEG caps with electrodes at set distances that can be easily applied at the bedside can also increase access to cEEG. Several EEG caps sized for neonates have become available for clinical use, with some having the ability to record using a full neonatal electrode configuration in the International 10–20 System, and some using a limited number of 6 or 10 electrodes, increasing the number of channels recorded compared to aEEG (60, 61). There is ongoing need for innovation to improve these caps, however, as one model of full montage EEG cap yielded uninterpretable recordings in 10% of neonates <35 weeks PMA and 52% of neonates ≥35 weeks PMA, with uninterpretable recordings more frequent with older, heavier infants with larger head circumferences (60). Wireless EEG devices can also be applied, and allow for wireless digital transmission of EEG recordings (60).

Another future direction of EEG includes expanding this form of monitoring *in utero*. When considering processes that begin before birth that predispose neonates to seizures, such as chorioamnionitis, placental insufficiency or chronic placental abruption, brain malformations, and neonatal onset genetic epilepsies, seizures can begin prior to delivery. When evaluating a neonate with seizures, a component of the pregnancy history includes abnormal movements, including repetitive movements sometimes interpreted as hiccups, which may indicate seizures occurring before the time of birth. Though these are clinically suspected to be seizures retrospectively, there is no current system to accurately diagnose or monitor for seizures *in utero*. There is ongoing research in fetal EEG in both animal models and in small studies involving limited electrodes in humans (62–65). Fetal EEG may be on the horizon to enhance early detection of seizures and may allow for improved early treatment of seizures using antiseizure medications that cross the placenta, or with administration of antiseizure medications at delivery.

## Conclusions

Continuous EEG is an essential tool for brain-centered care in the NICU. Assessment of the EEG background allows for improved prognostication in acute brain injury such as HIE. Continuous EEG is also the gold standard method for diagnosis and management of neonatal seizures and should be used to detect seizures in clinical conditions which portend a high risk for seizures, for differential diagnosis of paroxysmal events, and to assess response to treatment. Though there are clear benefits to cEEG, there are also significant equipment and personnel costs associated with cEEG implementation in a hospital system. Future applications of cEEG include centralized remote EEG interpretation to improve access to this resource, and fetal EEG to improve neuroprotection *in utero*.

## Author Contributions

ASK conceived of the idea and wrote the manuscript. CW provided critical feedback and contributed to and edited the manuscript. All authors contributed to the article and approved the submitted version.

## Funding

ASK is supported by a grant from the American Epilepsy Society and the Pediatric Epilepsy Research Foundation.

## Conflict of Interest

The authors declare that the research was conducted in the absence of any commercial or financial relationships that could be construed as a potential conflict of interest.

## Publisher's Note

All claims expressed in this article are solely those of the authors and do not necessarily represent those of their affiliated organizations, or those of the publisher, the editors and the reviewers. Any product that may be evaluated in this article, or claim that may be made by its manufacturer, is not guaranteed or endorsed by the publisher.
